# Alcohol use and spousal mental distress in a population sample: the nord-trøndelag health study

**DOI:** 10.1186/1471-2458-13-319

**Published:** 2013-04-09

**Authors:** Kamilla Rognmo, Fartein Ask Torvik, Espen Røysamb, Kristian Tambs

**Affiliations:** 1 Norwegian Institute of Public Health, Division of Mental Health, PO BOX 4404, Nydalen, Oslo N-0403, Norway; 2 Department of Psychology, University of Oslo, PO BOX 1094, Blindern, Oslo 0317, Norway

**Keywords:** Alcohol, Alcohol consumption, Alcohol abuse, Spousal mental distress, Anxiety, Depression, Population sample

## Abstract

**Background:**

It is a widely held notion that alcohol abuse is related to mental distress in the spouse. Research has substantiated this notion by showing a tendency for spouses of alcohol abusers to experience more mental distress than spouses of non-abusers. However, the picture seems to be more complex, as some results do not show a significant effect or even less mental distress among spouses of alcohol abusers with the highest alcohol consumption. The present study investigates the association between spousal mental distress and both a high consumption of alcohol and having experienced alcohol related problems.

**Methods:**

Norwegian population-based questionnaire data from the Nord-Trøndelag Health Study (HUNT 2) were analyzed. In total 11,584 couples were eligible for analysis. Alcohol consumption was measured by numerical indicators of alcohol amount and frequency of drinking, whereas alcohol-related problems (i.e. having been criticized for excessive drinking) were measured by the CAGE Alcohol Screening Questionnaire. Multivariate hierarchical regression analyses were performed.

**Results:**

Results revealed that alcohol consumption was significantly associated with a *decrease* in spousal mental distress, whereas alcohol-related problems were associated with an increase in spousal mental distress when adjusted for each other. Interaction effects indicated that couples discordant for drinking problems experienced more mental distress than spouses concordant for drinking problems.

**Conclusions:**

The results of our study indicate that alcohol-related problems constitute a clear risk factor for spousal mental distress. On the other hand, a high consumption of alcohol per se was related to lower levels of spousal mental distress, after adjusting for the alcohol-related problems perceived by the alcohol consumer him/herself. All effect sizes were small, but the trends were clear, challenging the notion that a high consumption of alcohol is exclusively and under all circumstances negative for the spouse.

## Background

It is widely believed that spouses of alcohol abusers suffer from poor mental health. This is a notion embraced by society in general, as well as in research communities and clinical practice [1,2]. The topic has been extensively studied, revealing both expected and unexpected effects. One population-based study found a small, but significant trend for female spouses of male at-risk drinkers to experience more mental distress than spouses of controls [3]. Another study found a three times higher risk of mood disorders and two times higher risk of anxiety disorders among female spouses of male alcohol abusers [4]. A study of newlywed individuals found frequent and heavy drinking among men, as well as marital and other problems resulting from the men’s drinking, to be associated with concurrent depression among their wives. Marital problems resulting from wives’ drinking were significantly associated with husbands’ concurrent depression. Longitudinally, marital problems due to husbands’ drinking were significantly associated with wives’ depression [1].

Despite an extensive range of studies showing a significant association, there are also several studies that did not find such significant associations – or else found results indicating a more complex relationship than previously assumed [5-8]. Schuckit and colleagues [5] found no elevated risk of psychiatric disorders among female spouses of male alcohol abusers. One explanation for the lack of significant effects may be that the sample mostly consisted of highly educated individuals. Socio-demographic factors may play a part in the association, providing a buffering effect against mental disorders.

Although it seems reasonable to assume that the risk of spousal mental distress increases with the alcohol consumption of the abusing partner, the relationship may not be that straightforward. Studies investigating how alcohol consumption among alcohol abusers may impact spousal psychiatric symptoms, have found a tendency for spouses of alcohol abusers with high consumption to have fewer psychiatric symptoms than spouses of alcohol abusers with lower consumption [6,7]. This result may indicate that stable heavy drinking, resulting in a higher overall consumption - as opposed to alternating between relapses and remitting periods – may serve some adaptive consequences for the family, through providing some stability in a stressful situation.

The presence of a substance use disorder also in the spouse of the alcohol abuser may impact the risk of mental distress. One study found a higher prevalence of psychiatric disorders only among spouses with co-occurring substance use disorders, but not among spouses without co-occurring substance use disorders [8]. However, concordant spousal alcohol consumption, even concordant heavy drinking, has also been found to predict marital satisfaction [9,10], which in turn is negatively related to mental distress [11]. Through impacting marital satisfaction, couples in which both spouses consume a lot of alcohol may experience less mental distress than couples where only one spouse drinks heavily.

Although it may be tempting to assume causal mechanisms behind the association between one partner’s’ alcohol abuse and the other partner’s’ mental distress, a third factor may be involved – spousal concordance across characteristics. This concordance may be attributed to several different mechanisms - assortative mating, mutual influence, or common pathogenic factors [12]. Rather strong observed spouse similarity for alcohol consumption is often assumed to reflect a tendency for people to marry persons with a consumption of alcohol similar to their own [12]. Assortative mating for alcohol consumption may confound the effect of alcohol consumption on spousal mental health, because one’s own alcohol abuse, independent of the spouse’s alcohol abuse, may be associated with one’s own mental health. In other words, a negative correlation between alcohol consumption in spouse A and mental health in spouse B could be a secondary result of the statistical relationship between alcohol consumption in spouse A and in spouse B and between alcohol consumption in spouse B and poor mental health in spouse B.

The inconsistent results from previous research clearly demonstrate the need for more evidence. First of all, less is known about how alcohol abuse in the female spouse relates to mental distress in the male spouse than the other way around. Second, the relationship may vary with educational level, mental distress in the alcohol abusing partner and alcohol abuse in the spouse in question. Third, there might be differential effects associated with a very high consumption of alcohol. By using a population-based sample, the present study aims at cross-sectionally investigating the relationship between high alcohol consumption and alcohol problems in men and women, and their spouses’ mental distress, while adjusting for factors identified by previous research to be influential.

## Methods

### Sample

During the years 1995 to 1997, the population aged 20 years or older in Nord-Trøndelag County, Norway, was invited to participate in a health screening survey, the Nord-Trøndelag Health Study (HUNT 2). The health study consisted of a health examination, including measuring blood pressure and cholesterol and a number of other analyses of the blood. Two questionnaires included items on health and illness, health behaviour, life-style, demography and other possible risk factors for health problems. Only the questionnaire data are used in the present study. The first questionnaire, Q1, was sent by mail together with the invitation letter and returned at the site of the health examination. The second questionnaire, Q2, was distributed during the health examination, and the participants were asked to complete it at home and return it by prepaid mail. The sample in the present study consists of participants aged 20-70, as participants over 70 years of age received a special questionnaire version, lacking one of the key items. Of the 77,659 invited individuals aged 20-70, 54,466 (70.1%) returned Q1 and 46,241 (59.5%) returned both Q1 and Q2. A detailed description of the full sample is available elsewhere [13]. The personal identification number assigned to all Norwegian citizens was used by the governmental statistics agency Statistics Norway to identify married or cohabiting couples. Only couples with complete data on all variables of interest after imputations were retained in the analyses, leaving 11,584 couples to be analyzed. These couples will be referred to as the nuclear sample from hereon. Mean age was 48.5 years (SD=11.2) in men and 45.7 years (SD=11.0) in women.

### Ethics

The HUNT-Study has been approved by the Regional Committee for Medical and Health Research Ethics for Central Norway and has been performed in accordance with the ethical standards laid down in the 1964 Helsinki declaration. All respondents gave their written consent for their data to be used for research purposes.

### Measures

#### Alcohol consumption (items in Q1)

The respondent was asked “Concerning alcohol, are you a non-drinker?” Next, alcohol consumption was measured by four items, of which one item asked how often the respondent usually drinks alcohol within a one month period. The response was given numerically. Amounts of consumption were measured by three items asking the respondent to numerically state how many units of beer, wine and liquor he/she usually drinks within a two-week period. One unit was defined as one glass of wine, beer or liquor. The four consumption items were combined into a summative scale. The Cronbach alpha was 0.67. The alcohol consumption index was trichotomized in each sex separately – categorizing the 95^th^ percentile with the lowest consumption in one category (low/moderate consumption), the 95^th^ to 98^th^ percentile in a high consumption category and the top 2% in a very high consumption category. Unlike a dichotomized measure the trichotomization is informative about the shape of the dose-response relationship. Since the validity of the alcohol consumption measure may have left something to be desired, we chose relatively high threshold values to be reasonably sure that the two highest categories represented factual-drinking/abuse.

#### Alcohol-related problems (items in Q2)

Alcohol-related problems were measured by the CAGE alcohol screening questionnaire [14] consisting of four items on whether the respondent had ever 1) felt the need to cut down on his/her drinking, 2) been criticized by others due to excessive drinking, 3) felt bad or guilty due to drinking, or 4) had a drink in the morning to steady nerves, get rid of a hangover or as an eye-opener. The items were coded “No” and “Yes”. For the sake of simplicity the items will be referred to as “Cut down”, “Criticized”, “Felt bad or guilty”, and “Eye-opener” from here on. Each CAGE item was used as an independent predictor of spousal mental distress because of the difference in content between the four items. This permitted examining which alcohol-related problems are more associated with spousal mental distress, and which are easier to live with for the spouse. For instance, being married to an individual who needs to have a drink in the morning to steady nerves may predict a worse outcome than being married to someone who has felt bad or guilty due to drinking.

#### Mental distress (items in Q1)

Symptoms of anxiety and depression were measured by 13 of the 14 items of the Hospital Anxiety and Depression Scale (HADS) [15]. Six of the items measured anxiety and seven measured depression. The items had four response categories, ranging from “not present” to “highly present”, and the reference period was the week prior to responding to the questionnaire. The HADS depression items only measure absence of positive emotionality, not negative emotionality. Therefore, the HADS was supplemented by the CONOR Mental Distress Index (CMD): In the last two weeks, have you felt: “Confident and calm?”, “Happy and optimistic?”, “Nervous and restless?”, “Troubled by anxiety?”, “Irritable?”, “Down/depressed?”, “Lonely?” (response categories: “no”, “a little”, “a good amount”, “very much”). CMD is described in detail elsewhere [16]. The HADS and the CMD scores were summed to a global mental distress index and standardized. The HADS and the combined HADS/CMD instrument correlated 0.96 for men and 0.97 for women. By adding the CMD to the HADS, the Cronbach alpha increased from 0.86 to 0.90 for men and from 0.83 to 0.91 for women.

#### Demographic data

Data on age, with whole years as measurement unit, were obtained from a public registry. Education was scored as one of four categories ranging from primary school to four years or more at college/university.

### Treatment of missing values

SPSS Missing Value Analysis (MVA), Expectation Maximization (EM) was used to impute values separately for the various set of items (mental distress, alcohol consumption, CAGE). Imputations were made for respondents with valid data for a minimum of 50% of the items of each set of items. The proportion of respondents with non-complete data was reduced from 17.8% to 1.0% for mental distress. Most of these had only one or two missing items. Prior to imputations of the alcohol consumption items, the respondents having reported to be abstainers and who had not completed the alcohol consumption items were scored zero on all the items. In cases where some of the consumption items were blank and other consumption items had valid responses higher than zero, we assumed that blank responses signified no consumption for those beverages and replaced these missing values by zero. For instance, if a person had reported five units of beer and seven units of wine and left liquor open, this was assumed to mean “no liquor”. This recoding (abstainers and blanks in combination with responses >0) reduced incomplete alcohol consumption data from 51.5% to 6.5%. Subsequently, the alcohol consumption items were imputed for respondents with valid data for a minimum of 50% of the items, which reduced missing values from 6.5% to 4.6%. Imputations of CAGE only reduced missing values from 8.5% to 7.7%. Due to the small number of records gained by imputing CAGE, we chose to replace missing values for the individuals who had scored less than the average on the alcohol consumption index with the response indicating absence of alcohol-related problems on the CAGE items. This reduced incomplete/missing CAGE data from 7.7% to 0.8%.

### Statistical analyses

Multivariate hierarchical regression analyses (SPSS, Regression, Linear) were run stratified by gender, investigating the predictive value of male and female index persons’ alcohol consumption and alcohol-problems on their spouses’ mental distress. To investigate deviations from linearity for the effect of the trichotomized alcohol consumption variable on spousal mental distress, curve estimation regression analyses were performed. The R^2^ of the functions allowing curve linearity was not higher than the R^2^ of the linear regression analysis, supporting the usage of a trichotomized alcohol consumption variable. Spearman rank-order correlations were run for the index persons’ alcohol predictor and spousal mental distress, giving crude estimates of the associations. In the hierarchical regression analyses, spousal age and spouses’ and index persons’ educations were entered in the first model (model 1). In model 2, alcohol consumption was added to the model. To observe the predictive effect of the CAGE items without adjusting for alcohol consumption, the CAGE items were entered together with the demographic variables from the first model in model 3. In model 4, alcohol consumption and the CAGE items were entered together with the demographic variables to observe the effects of each alcohol predictor when adjusted for the other alcohol predictors.

**Figure 1:**
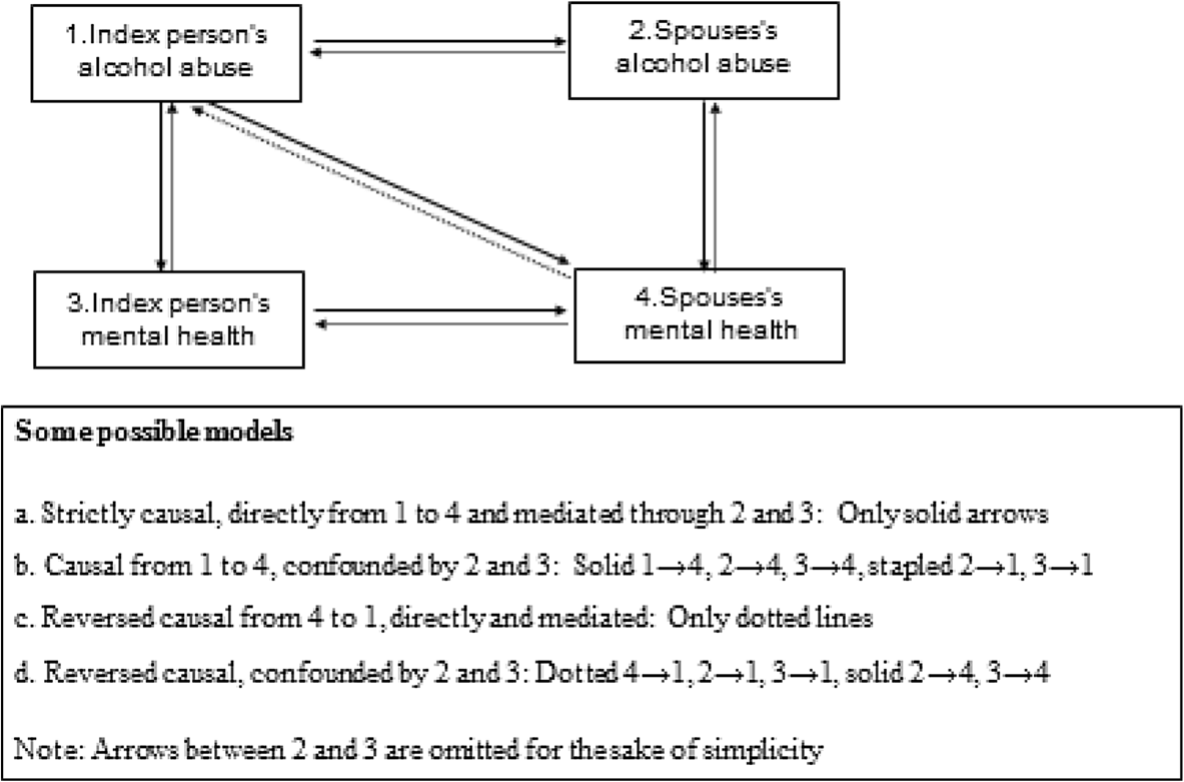
Some possible causal pathways between index persons’ and spouses’alcohol abuse and mental health.

The effect of alcohol consumption/problems in the index person on mental health in the spouse may be somewhat confounded by the index person’s mental distress, because the index person’s mental distress may have an impact both on their own alcohol consumption/problems and on the spouse’s mental health. Likewise the spouse’s own alcohol consumption or alcohol-related problems may affect both the index person’s alcohol consumption/problems and the spouse’s mental health, and accordingly act as a confounder. Just as likely, however, these variables (the index person’s mental distress and the spouse’s alcohol consumption and alcohol-related problems) may act as mediators. That is, the index person’s alcohol consumption/problems cause distress in the index person and increased alcohol consumption/problems in the spouse, and these again cause distress in the spouse. Research has shown that alcohol abuse most likely causes within-person mental distress, rather than the other way around [17], suggesting that the spouse’s mental distress may act more as a mediator than as a confounder. Research regarding the causality of spousal concordance for alcohol consumption implies that both assortative mating and spousal convergence over time are involved [18], suggesting that alcohol use and alcohol-related problems may both confound and mediate the effect of index persons’ alcohol consumption/problems on spouses’ mental distress. The possible causal pathways between index persons’ and spouses’ alcohol variables and mental distress are presented in Figure 1. In conclusion, whereas model 4 may be somewhat under-adjusted, model 5, including index person’s mental distress and spousal alcohol variables, may be over-adjusted because it probably adjusts for mediator effects.

Spousal and own alcohol consumption and alcohol problems may act as effect modifiers for each other. Thus, interaction terms between index persons’ alcohol variables (alcohol consumption and each of the CAGE items), and between index person and spouse alcohol variables were specified. Demographic characteristics of the spouse or the couple and index person mental distress may also moderate the effects of the alcohol variables. We tested for interaction effects between index person alcohol variables and the following variables: spousal age, average couple education, and index person mental distress. All interaction effects were adjusted for all variables included in model 4.

## Results

### Descriptive statistics

**Figure 2:**
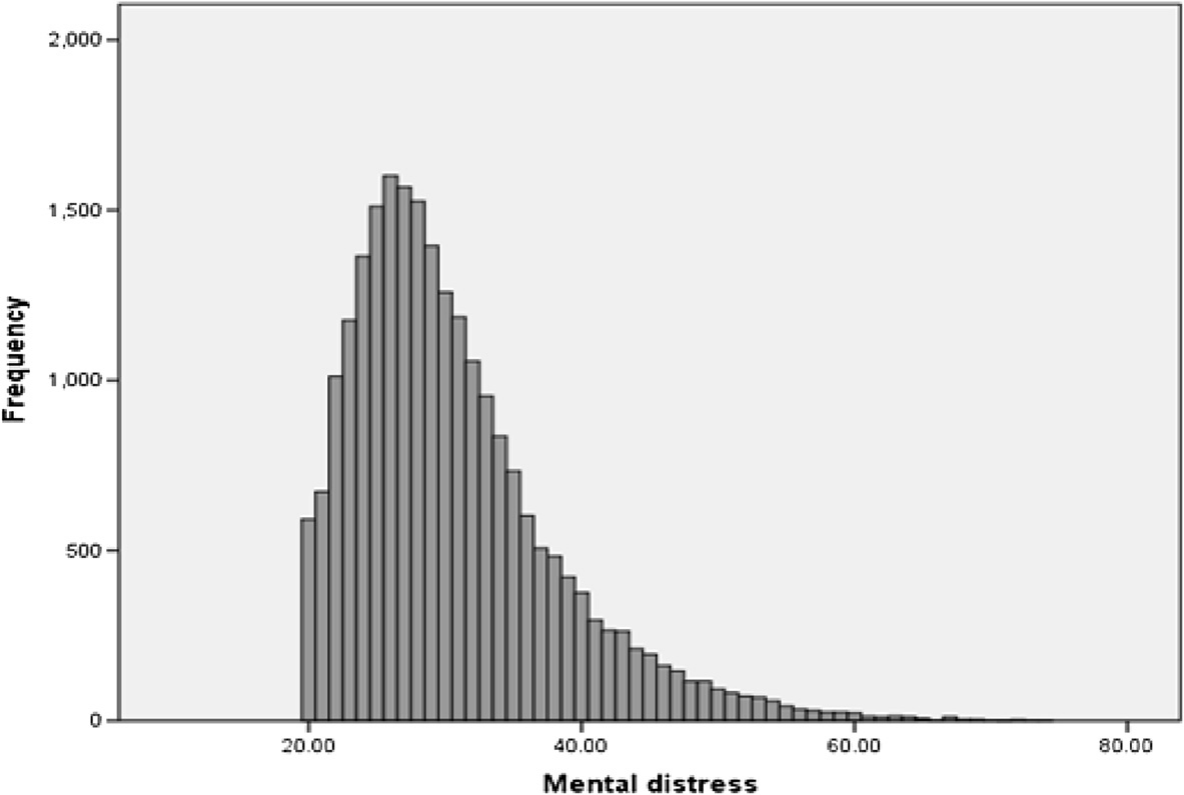
Histogram with a normal probability plot of mental distress for women and men combined.

The distribution of the outcome measure, mental distress, is presented in Figure 2, collapsed across genders. It deviates somewhat from a normal distribution, with skewness 1.31 and kurtosis 2.29.

Mean alcohol consumption (sum of units of alcohol drunk within a two-week period) for male respondents was 4.3 (range 0-50) and 64.3% reported drinking less than the mean. The top 15% reported drinking 8.0 units or more. Mean alcohol consumption for female respondents was 2.3 (range 0-40) and 65.8% reported a consumption lower than the mean. The top 15% reported drinking 4.2 units or more. Mean values for males and females in the different alcohol consumption groups are presented in Table 1.

**Table 1:** Mean and standard deviations of total numbers of reported glasses of beer, wine and liquor normally consumed within a two-week period for women and men in the low, high and very high consumption groups

	**Men**		**Women**	
	**M**	**SD**	**M**	**SD**
*Low alcohol consumption*	3.6	3.4	1.8	2.0
*High alcohol consumption*	15.0	3.8	8.7	2.1
*Very high alcohol consumption*	22.6	7.5	14.2	5.3

Spearman rank-order correlations between the various alcohol measures are presented in Table 2. Table 2 also contains bivariate associations between alcohol consumption, the CAGE items and mental distress for both index persons and spouses. Unadjusted, male alcohol consumption was not significantly associated with spousal mental distress. All the CAGE items for male index persons were significantly associated with female spousal mental distress. The unadjusted association between female index persons’ alcohol consumption and male spousal mental distress was just significant, whereas two of the CAGE items, “Criticized” and “Felt bad or guilty” were significantly associated with male spousal mental distress. The direction of the relationship with spousal mental distress was negative for alcohol consumption and positive for CAGE. The within-subject correlation between alcohol consumption and mental distress was non-significant for both female and male respondents, whereas the CAGE items were moderately correlated with within-subject mental distress – with correlations ranging from .09-.15 and .03-.11 for males and females, respectively.

**Table 2:** **Spearman rank correlations between alcohol variables and mental distress for index persons and spouses**^**a**^

	**1**	**2**	**3**	**4**	**5**	**6**	**7**	**8**	**9**	**10**	**11**	**12**
1: Spousal mental distress		-.02**	.02	.02**	.04***	.01	.25***	.02*	.13***	.13***	.15***	.07***
2: Alcohol consumption	-.02		.22***	.07***	.16***	.04***	-.01	.32***	.13***	.03***	.05***	.00
3: Cut down	.02*	.21***		.36***	.41***	.19***	.09***	.11***	.11***	.03***	.06***	.01
4: Criticized	.04***	.14***	.45***		.28***	.25***	.07***	.02**	.04***	.01	.03**	.02**
5: Felt bad or guilty	.04***	.11***	.44***	.45***		.14***	.11***	.06***	.10***	.07***	.13***	.03**
6: Eye-opener	.02**	.06***	.22***	.24***	.19***		.03***	.01	.02	.00	.02*	.04***
7: Index mental distress	.25***	.02	.13***	.13***	.15***	.07***		-.02	.03*	.04***	.04***	.03**
8: Spouse alcohol consumption	-.00	.32***	.14***	.04**	.05***	.00	-.02*		.21***	.14***	.11***	.06***
9: Spouse Cut down	.09***	.10***	.11***	.03***	.07***	.01	.02	.22***		.46***	.44***	.22***
10: Spouse Criticized	.07***	.02*	.04***	.01	.03***	.02	.02**	.08***	.38***		.45***	.24***
11: Spouse Felt bad or guilty	.10***	.07***	.11***	.07***	.14***	.03**	.04***	.17***	.41***	.28***		.19***
12: Spouse Eye-opener	.04***	.01	.02	.00	.02**	.04***	.01	.04***	.19***	.24***	.14***	

^a^ Estimates above the diagonal are female index persons and male spouses and below the diagonal are male index persons and female spouses.

* p.<.05.

** p.<.01.

*** p.<.001.

### Results of the hierarchical regression analyses

The predictor variables were entered block-wise into the regression analyses. The analyses were stratified by sex. The results of the hierarchical regression analysis of male index persons and female spouses are presented in Table 3, followed by the results for female index persons and male spouses in Table 4.

**Table 3:** Hierarchical regression analyses of the effect of male index persons’ alcohol consumption and CAGE items on female spouses’ mental distress

		**Scaling of variable**	**B**^**a**^	**95% CI (B)**	**beta**^**a**^	**p**	**adj. R **^**2**^	**p for R **^**2 **^**change**^**d**^
Model 1:							.015	.000
	Age spouse	Years	.006	.004, . 008	.065	.001		
	Education	1 – 4^b^	-.034	-.051, -.018	-.043	.001		
	Education spouse	1 – 4 ^b^	-.045	-.062, -.027	-.056	.001		
Model 2:							.015	.169
	Age spouse	Years	.006	.004, .008	.066	.001		
	Education	1 – 4 ^b^	-.033	-.050, -.017	-.042	.001		
	Education spouse	1 – 4 ^b^	-.045	-.062, -.027	-.056	.001		
	Alcohol consumption	1 – 3 ^c^	-.043	-.105, .018	-.013	.169		
Model 3:							.017	<.000^e^
	Age spouse	Years	.007	.005, .008	.070	.001		
	Education	1 – 4 ^b^	-.033	-.050, -.017	-.042	.001		
	Education spouse	1 – 4 ^b^	-.046	-.063, -.028	-.057	.001		
	Cut down	0, 1	-.009	-.080, .061	-.003	.793		
	Criticized	0, 1	.065	-.005, .135	.020	.070		
	Felt bad or guilty	0, 1	.045	.036, .154	.034	.002		
	Eye-opener	0, 1	.084	-.039, .206	.013	.182		
Model 4:							.017	<.000
	Age spouse	Years	.007	.005, .008	.071	.001		
	Education	1 – 4 ^b^	-.032	-.048, -.015	-.040	.001		
	Education spouse	1 – 4 ^b^	-.045	-.063, -.028	-.057	.001		
	Alcohol consumption	1 – 3 ^c^	-.069	-.132, .006	-.020	.032		
	Cut down	0, 1	.002	-.069, .074	.001	.949		
	Criticized	0, 1	.068	-.002, .139	.021	.056		
	Felt bad or guilty	0, 1	.095	.036, .154	.034	.002		
	Eye-opener	0, 1	.086	-.037, .209	.013	.169		
Model 5:							.084	<.000
	Age spouse	Years	.006	.005, .008	.068	.001		
	Education	1 – 4 ^b^	-.021	-.037, -.005	-.026	.012		
	Education spouse	1 – 4 ^b^	-.042	-.058, -.025	-.052	.001		
	Alcohol consumption	1 – 3 ^c^	-.085	-.149, .-021	-.025	.010		
	Cut down	0, 1	-.076	-.145, -.007	-.023	.032		
	Criticized	0, 1	.034	-.034, .102	.010	.325		
	Felt bad or guilty	0, 1	.002	-.056, .059	.001	.951		
	Eye-opener	0, 1	.018	-.100, .137	.003	.763		
	Mental distress	standardized	.250	.231, .270	.228	.001		
	Alcohol consumption spouse	1 – 3 ^b^	-.011	-.069, .047	-.004	.710		
	Cut down spouse	0, 1	.347	.215, .478	.054	.001		
	Criticized spouse	0, 1	.296	.124, .467	.033	.001		
	Felt bad or guilty spouse	0, 1	.327	.236, .418	.071	.001		
	Eye-opener spouse	0, 1	.389	.123, .655	.027	.004		
	N=11,584 couples							

^a^ B= unstandardized regression coefficient, beta=standardized regression coefficient.

^b^ 1= primary school, 4 = ≥years at university/college.

^c^ 1= low/moderate, lower 95%, 2 = high, 95-98%, 3= very high, top 2%.

^d^ P-value of the F-test of the R^2^ change compared to former model.

^e^ Compared to model 1.

Spouses’ own responses.

**Table 4:** Hierarchical regression analyses of the effect of female index persons’ alcohol consumption and CAGE items on male spouses’ mental distress

		**Scaling of variable**	**B**^**a**^	**CI (B)**	**beta**^**a**^	**p**	**adj. R**^**2**^	**p for R **^**2 **^**change**^**d**^
Model 1:							.008	.000
	Age spouse	Years	.002	.000, .003	.022	.024		
	Education	1 – 4^b^	-.020	-.035, -.004	-.027	.015		
	Education spouse	1 – 4 ^b^	-.048	-.063, -.033	-.067	.001		
Model 2:							.008	.101
	Age spouse	Years	.002	.000, .004	.023	.017		
	Education	1 – 4 ^b^	-.019	-.034, -.003	-.026	.020		
	Education spouse	1 – 4 ^b^	-.047	-.062, -.032	-.066	.001		
	Alcohol consumption	1 – 3 ^c^	-.043	-.095, .008	-.015	.101		
Model 3:							.010	.000^e^
	Age spouse	Years	.002	.000, .004	.025	.012		
	Education	1 – 4 ^b^	-.020	-.036, -.004	-.028	.012		
	Education spouse	1 – 4 ^b^	-.048	-.063, -.034	-.067	.001		
	Cut down	0, 1	-.038	-.158, .082	-.006	.538		
	Criticized	0, 1	.165	.004, .327	.020	.045		
	Felt bad or guilty	0, 1	.153	.069, .237	.037	.001		
	Eye-opener	0, 1	-.067	-.332, .198	-.005	.620		
Model 4:							.010	.000
	Age spouse	Years	.002	.001, .004	.027	.007		
	Education	1 – 4 ^b^	-.019	-.035, -.003	-.026	.018		
	Education spouse	1 – 4 ^b^	-.047	-.062, -.032	-.066	.001		
	Alcohol consumption	1 – 3 ^c^	-.063	-.116, -.010	-.022	.020		
	Cut down	0, 1	-.013	-.135, .109	-.002	.837		
	Criticized	0, 1	.163	.001, .324	.020	.048		
	Felt bad or guilty	0, 1	.160	.076, .244	.039	.001		
	Eye-opener	0, 1	-.069	-.334, .196	-.005	.610		
Model 5:							.093	.000
	Age spouse	Years	.002	.000, .004	.024	.010		
	Education	1 – 4 ^b^	-.010	-.025, -.005	-.014	.187		
	Education spouse	1 – 4 ^b^	-.039	-.053, -.024	-.054	.001		
	Alcohol consumption	1 – 3 ^c^	-.086	-.140, .-033	-.031	.002		
	Cut down	0, 1	-.123	-.240, -.006	-.021	.040		
	Criticized	0, 1	.124	-.031, .276	.015	.116		
	Felt bad or guilty	0, 1	.005	-.076, .086	.001	.902		
	Eye-opener	0, 1	-.130	-.384, .124	-.009	.314		
	Mental distress	Standardized	.204	.188, .220	.225	.001		
	Alcohol consumption spouse	1 – 3 ^b^	.027	-.027, .081	.009	.326		
	Cut down spouse	0, 1	.176	.113, .238	.059	.001		
	Criticized spouse	0, 1	.160	.098, .221	.054	.001		
	Felt bad or guilty spouse	0, 1	.230	.178, .281	.091	.001		
	Eye-opener spouse	0, 1	.221	.114, .329	.037	.001		
	N=11,584 couples							

^a^ B= unstandardized regression coefficient, beta=standardized regression coefficient.

^b^ 1= primary school, 4 = ≥years at university/college.

^c^ 1= low/moderate, lower 95%, 2 = high, 95-98%, 3= very high, top 2%.

^d^ P-value of the F-test of the R^2^ change compared to former model.

^e^ Compared to model 1.

Spouses’ own responses.

### Male index persons – female spouses

The results from model 1, where the demographic variables were entered together, showed that spousal age and spousal and index person education were significantly related to spousal mental distress. In model 2, index person alcohol consumption was entered into the model but did not significantly predict spousal mental distress. In model 3, index person alcohol consumption was removed from the model, and replaced by index persons’ CAGE responses. Only one CAGE item, “Felt bad or guilty” significantly predicted spousal mental distress. In model 4, both index person alcohol consumption and CAGE were entered simultaneously into the model. When adjusting for demography and CAGE, alcohol consumption significantly predicted a *decrease* in spousal mental distress. “Felt bad or guilty” was still the only CAGE item significantly predicting an increase in spousal mental distress. In model 5 spouse alcohol variables and index person mental distress were added to the model, to explore potential mediation or confounding. The negative effect of index persons’ alcohol consumption increased in model 5, indicating that some of the effect of alcohol consumption was suppressed in models 1-4. The effect of the CAGE item “Felt bad or guilty” seemed to be completely mediated or confounded by the new variables entered in model 5 and lost its significant effect. “Cut down” was the only significant CAGE item in model 5, predicting a decrease in spousal mental distress. The remaining CAGE estimates decreased in magnitude in model 5. Spouses’ own alcohol consumption was unrelated to their mental distress, when adjusting for all the variables in block 5. All four spousal CAGE items were highly predictive of spouses’ own mental distress.

### Female index persons – male spouses

The relationship between female index persons’ alcohol consumption and alcohol-related problems, measured by CAGE, and their male spouses’ mental distress was investigated with the same hierarchical procedure as reported above. Model 1 showed that spousal age and spousal and index person education were significantly related to spousal mental distress. In model 2, alcohol consumption was entered into the model, but did not significantly predict spousal mental distress. In model 3, alcohol consumption was replaced by the index persons’ CAGE items, of which the items “Criticized” and “Felt bad or guilty” significantly predicted an increase in spousal mental distress. In model 4, index person alcohol consumption and CAGE were entered simultaneously. When adjusting for demography and index person CAGE, alcohol consumption significantly predicted a decrease in spousal mental distress. The CAGE items “Criticized” and “Felt bad or guilty” still significantly predicted an increase in spousal mental distress in model 4. In model 5, the effect of index person alcohol consumption increased, and still significantly predicted decreasing spousal mental distress. Two index person CAGE items also significantly impacted the outcome. “Cut down” was negatively associated with and “Criticized” positively associated with spousal mental distress. All CAGE estimates, except “Cut down” decreased in magnitude in model 5. Spouses’ own alcohol consumption was unrelated to their mental distress, whereas all spousal CAGE items were positively related to their own mental distress.

### Interaction effects

Interaction terms between index persons’ alcohol variables, between index persons’ and spouses’ alcohol variables, between index persons’ alcohol variables and mental distress, and between index persons alcohol variables and the demographic variables were specified. In total, 49 interaction analyses were run. The significance level was corrected according to the Bonferroni formula, rendering the alpha level at 0.001. This resulted in two significant interaction effects – between 1) female index persons’ and male spouses’ alcohol consumption (*p*=.001), and 2) “Cut down” in male index persons and “Criticized” in female spouses (*p*<.001). Two effects were approaching significance, after Bonferroni correction - 3) “Criticized” in male index persons and “Eye-opener” in female spouses (*p*=.008); and 4) “Felt bad or guilty” and mental distress in female index persons (*p*=.009).

Simple effects analyses in stratified samples showed 1) that the negative (protective) effect of alcohol consumption among female index persons was stronger for spouses consuming high amounts (*B*=-.298, *p*=.001) and very high amounts (*B*=-.200, *p*=.012) than for spouses with a low/moderate consumption (*B*=-.024, *p*=.495. 2) The negative effect of “Cut down” in male index persons was stronger for female spouses who themselves had been “Criticized” (*B*=-.679, *p*=.015) than for spouses who had not been “Criticized” (*B*=.076, *p*=.224). 3) The positive (risk) effect of “Criticized” in male index persons on spousal mental distress tended to be stronger for female spouses who had not had an “Eye-opener” to steady nerves (*B*=.078, *p*=.029), than for female spouses who had had an “Eye-opener” (*B*=-.732 *p*=.421). 4) The positive effect of mental distress in female index persons tended to be stronger when the index person themselves had not “Felt bad or guilty” (*B*=.466, *p*=.000) than when the index person had “Felt bad or guilty” (*B*=.211, *p*=.026).

## Discussion

The inconsistent findings from previous research on the relationship between one spouse’s alcohol abuse and the other spouse’s mental health indicate that the relationship is more complex than previously assumed. This study seconds this interpretation, as the results suggest that alcohol consumption was related to less spousal mental distress, whereas alcohol-related problems were either unrelated to spousal mental distress or related to higher levels of spousal distress. Bivariate analyses of the association between alcohol consumption and spousal mental distress were not sufficient to produce this result, whereas a slightly *protective* effect of alcohol consumption appeared when the acknowledged problems associated with alcohol abuse - in terms of CAGE scores - were adjusted for.

*Alcohol-related problems*, measured by CAGE, were in general related to an increase in spousal mental distress, although the specific type of problem that significantly predicted the outcome varied according to which variables were entered into the model. The strongest effects were found in model 4, where the effects of CAGE were adjusted for demography and alcohol consumption. The results are in accordance with results reported by Tempier et al. [3] who found a small increase in spousal mental distress associated with index persons with a minimum of two positive CAGE responses. In our study, both female and male spouses of persons who had felt bad or guilty due to excessive drinking had overall significantly higher mental distress than other spouses. Having felt bad or guilty due to excessive drinking involves a certain degree of realization that the drinking is causing problems either for oneself or for others, and as such may be indicative of long-term -or serious drinking problems. Also, male spouses of female index persons who had been criticized for excessive drinking had significantly higher scores on mental distress. Most likely, one of the persons having criticized the alcohol consumption is the spouse him/herself. A lack of cultural acceptance for a high consumption of alcohol among women may cause male spouses to criticize female spouses’ alcohol consumption more readily. However, the graver social stigma of female alcohol abuse may also cause male spouses to evade criticizing their spouses’ consumption. Thus, the item “Criticized” may in fact reflect a very high consumption, and possibly be highly suggestive of alcohol abuse among female index persons.

Including the variables in model 5 may represent an over-adjustment of the effects of the predictor variables, but is informative as to the magnitude of the additional variables’ joint mediating and/or confounding effects on the effects of index person alcohol consumption and problems. The effects of the CAGE items decreased for both male and female index persons when adding spousal alcohol variables and index person mental distress in model 5, indicating partial mediation or confounding by at least some of these additional variables. The decrease may be particularly due to a relatively strong effect (as judged by the standardized beta values) of the index persons’ mental distress, which seems to “take over” the effect of the worry expressed by the CAGE items. We find it likely that the observed mental distress mediates, rather than confounds, the effects of the CAGE items, implying that the estimates in model 5 are somewhat over-adjusted. On the other hand, the estimates in model 4 may be slightly under-adjusted, suggesting that the realistic values of the estimates are somewhere between those from model 4 and those from model 5.

The estimated effects of each of the CAGE items must be conceived as fractions of the total effect of CAGE, most of them down-adjusted because of inter–item correlations. However, the fact that the CAGE items behaved rather differently in their relationship with spousal mental distress after adjusting for alcohol consumption justifies the usage of each item as a single predictor. Using the CAGE items in the traditional manner – by using a cut off at two positive CAGE responses – would disguise this difference in directionality of the CAGE items, giving a less informative portrait of the relationship between our alcohol variables and spousal mental distress.

Spouses of index persons having felt the need to “Cut down” had significantly less mental distress compared with spouses of index persons who had not felt such a need (model 5). Also, the “Eye-opener” item of female index persons tended to be associated with less mental distress, although not significantly so. The other CAGE items seemed to be related to spousal mental distress, although at levels varying from non-significant to significant, and only significantly in model 4.

The results for *alcohol consumption* from model 4 indicate that there also may be constructive factors associated with drinking alcohol, after adjusting for alcohol-related problems. Our data were not based on diagnosis, and therefore we cannot be absolutely sure that the individuals categorized as high and very high consumers in fact have a problematic relationship to alcohol. By categorizing only the top 5% in a high and very high consumption group, most of the persons in these groups are probably abusers, as the 12-month prevalence rate of alcohol use disorders in Norway has been observed to be higher (16.4% for men and 6.0% for women) [19]. However, there will also most likely be high and very high consumers in our study who do not qualify for an alcohol abuse diagnosis, and consequently we cannot state positively that our consumption measure is indicative of alcohol abuse as such. Rather, our results indicate that altogether a high consumption is weakly related to good spousal mental health. This interpretation may be seconded by results from the Schuckit et al. [5] study. Although not statistically significant, Schuckit and colleagues found a tendency for spouses of alcohol abusers to have fewer psychiatric symptoms than spouses of non-abusers. A high consumption of alcohol may be related to other third factors not measured in this study. For instance, a high consumption of alcohol may involve more social activities, and being socially active may be protective against mental distress [18]. Also, drinking large quantities of alcohol may be related to a tendency to enjoy oneself - of being a bon vivant - which possibly may be constructive for the spouse.

Concordance for high alcohol consumption has previously been found to indicate high marital satisfaction [9], which in turn may cause less mental distress [11]. The significant interaction effects indicated that concordance for high alcohol consumption was related to significantly less mental distress and discordance for alcohol consumption to increased mental distress. Female index persons’ alcohol consumption showed a stronger negative (protective) relationship with mental distress among male spouses with high and very high alcohol consumption than among spouses with a low/normal consumption. Similar results were found for male index persons having felt the need to cut down on drinking and female spouses having been criticized for drinking. Female spousal mental distress tended to be more increased by the male index person having been criticized in spouses who had *not* had an “Eye-opener” than in spouses having had an “Eye-opener”, although this trend did not reach significance after a Bonferroni correction. These results may indicate that spousal discordant drinking patterns may increase the risk of experiencing mental distress, whereas spousal concordant drinking patterns may to a certain extent protect against mental distress, which may or may not be a function of marital satisfaction.

Previous research has suggested that the relationship between alcohol abuse and spousal mental distress may be dependent upon educational level [5]. Higher education is related to higher alcohol consumption, but also to lower frequency of binge drinking [20]. Mental disorders are more prevalent among individuals with low socioeconomic status [21]. We found no significant interaction effects suggesting a moderator effect of educational level.

Our results on alcohol *consumption* are in contrast to the results of the study by Maes et al. [12], who found significant cross-assortment between alcohol abuse in one partner and anxiety or depression in the other. Such cross-assortment implies a correlation between trait A in spouse 1 and trait B in spouse 2 after the correlation due to assortment for other correlated variables has been partialled out [12]. Our results on alcohol-related *problems* are consistent with those of Maes et al. Different methods may explain the partial discrepancy. The Maes et al. study was based on lifetime diagnoses obtained by structured interview, whereas our study examined current self-reported consumption, lifetime alcohol problems, and symptoms of anxiety and depression assessed by questionnaire. The study by Dawson et al. [4], who also found a higher risk of anxiety and depression among female spouses of male alcohol abusers, identified spouses of alcohol abusers by asking the respondents to indicate whether or not their spouses had an alcohol use disorder. Only a small fraction of their sample reported having husbands with alcohol use disorders, incongruent with the prevalence numbers of alcohol use disorders in the United States. This may imply that their results only apply for spouses of the most seriously afflicted alcohol abusers, which may be why such a high risk of spousal anxiety and depression was found. An even more important reason for the large effect size reported by Dawson et al. may have been a misclassification of the alcohol cases correlated by the outcome measure. That is, depressed persons may be more inclined to characterize their partners as alcohol abusers than are mentally healthy persons.

The sizes of the effects of a high and very high consumption on spousal mental distress in our study were quite small and probably would not have been detected as significant in a sample much smaller than ours. Despite the small effects, the tendency regarding positive aspects of a high consumption was clear, challenging the notion that a high alcohol consumption is exclusively related to negative aspects for the spouses. Due to our study’s large sample and precise estimates, this finding adds to the existing literature by showing that once the variation associated with problems related to drinking is accounted for, alcohol consumption seems either directly or indirectly to be related to good spousal mental health.

### Methodological considerations

The existing literature on the relationship between one spouses’ alcohol abuse and the other spouses’ mental distress has for the most part only investigated the relationship between male alcohol abusers and female spouses. Also, several of the previous studies have been based on samples of limited generalizability. The present study is based on data from both male and female spouses, from a large sample, representative of the Norwegian adult population, implying a high generalizability of our results.

However, there are methodological limitations to our study. The response rate for the individuals having returned both Q1 and Q2 (59.5%) may have caused a selection bias. However, a recent attrition study of the HUNT 2 sample showed that high alcohol consumption in a previous HUNT study only predicted non-participation in HUNT 2 moderately well (OR= 1.27 for the top 3% consumption) [22]. Also, even highly selective non-participation only seems to moderately influence associations between variables [23], giving reason to believe that our estimates have not been severely affected by non-participation.

The lack of diagnostic measures rendered us incapable of positively identifying alcohol abusers among the index persons. As shown by the mean values of the high and very high consumption groups, the consumption reported may not be indicative of alcohol use disorders. However, alcohol consumption is usually underreported in population studies [24], making it feasible to assume that the actual consumption is considerably higher than reported. The degree to which underreporting leads to misclassifications depends upon whether or not the underreporting is systematic - that is, whether the amount of under-reporting correlates highly with the real consumption, such that most people report, say, half their real consumption - or non-systematic. The distribution of the consumption in our sample corresponds well with distributions normally observed in alcohol consumption research, in which approximately 2/3 of the population reports drinking less than the average consumption and 15% reports drinking more than twice that of the average [25]. This suggests that the underreporting is systematic, and that the high and very high consumption groups in fact primarily include high and very high consumers – who for the large part will be individuals with alcohol use disorders. By choosing strict criteria for what is considered high and very high consumption, there is a much higher probability of misclassifying a real case as a non-case than of misclassifying a non-case as a case. To avoid substantial attenuation of the results, keeping the case groups relatively free from false positives is much more important than is avoiding some pollution of the large non-case group by (a relative low fraction of) false negatives. Therefore, the choice of strict criteria defining the top five percentile as high and very high consumers with potential alcohol problems is methodologically sound.

Alcohol consumption was measured as current consumption, whereas alcohol-related problems were measured as lifetime problems. The outcome, mental distress, was also measured as current distress. Current disorders are more predictive of co-occurring problems in the spouse [1], which may have deflated the observed association between alcohol-related problems and spousal mental distress. Furthermore, individuals abstaining from alcohol were asked to skip the CAGE, which may have caused previous alcohol abusers to skip this measure given that they were abstaining at the time of the survey. It was not possible to separate steady heavy drinkers from binge drinkers in this data material. Previous research has found that spouses of steady heavy drinkers experience less mental distress than spouses of binge drinkers – although the total alcohol consumption of steady heavy drinkers in general is higher than that of binge drinkers [6]. Being able to distinguish effects of steady heavy drinking from effects of binge drinking on spousal distress would have added information to our results.

The mental distress index was composed of two separate mental health measures, the HADS and the CMD, which has not previously been used in a combined version, and of which only the first has previously been validated. However, we still judged the face validity of the combined index to be better than that of the HADS alone, because the HADS depression items seem not to include negative emotionality. The correlation between the HADS scores and the scores from the combined index was 0.96 for men and 0.97 for women, so clearly the content of the HADS was not radically changed after the inclusion of the CMD items. Also, the internal consistency of the measure increased after including the CMD. Accordingly, even if our measure was not strictly validated, we must assume that the validity was as least as good as for the HADS instrument.

Our design is not suited to decide about the causal direction between alcohol abuse and spousal mental distress. Although not evidence-based, we would judge a causal direction from alcohol abuse in one of the spouses to mental distress in the other as more plausible than the reversed causal pathway, but there are probably also people who drink to drown problems related to their spouse’s’ poor mental health.

Finally, and related to the problem of unknown causality, our results could be confounded by effects of assortative mating, primarily for alcohol consumption, for which there is a strong spouse correlation. The association between alcohol abuse in spouse A and mental health in spouse B could partly reflect assortative mating for alcohol abuse together with a relationship between own alcohol abuse and own mental health. However, recent results based on HUNT data suggest that most of the observed spouse similarity for alcohol consumption reflects convergence during the spouses’ life together rather than assortative mating. Only a minor part of the spouse similarity seems to have been present at the time the spouses started to see each other [26]. Also, the association between own alcohol abuse and mental health is moderate (Table 1), implying that an important confounding by assortative mating is unlikely.

## Conclusions

The results of our study indicate that alcohol consumption and alcohol-related problems partly predict different spousal outcomes. Alcohol-related problems constitute a clear risk factor for spousal mental distress. When disentangling alcohol-related problems perceived by the alcohol consumer him/herself from other effects of alcohol consumption, it appears that besides the adverse consequences of spousal drinking problems, there is a protective effect of alcohol consumption against spousal mental distress. Although the effect sizes were small, the trend was clear – the higher the consumption, the less mental distress was experienced by the spouse. Explanations for this result might partly be found in non-investigated third factors. Drinking alcohol may, for instance, be related to a tendency to enjoy oneself or to be socially active. Apart from the primarily adverse effect of problematic alcohol use on spousal mental health, differences in alcohol use between the spouses represents an additional risk factor, whereas spouse similarity may buffer negative effects of spousal alcohol consumption.

## Competing interest

The authors declare that they have no competing interests.

## Authors’ contributions

KR was responsible for the design, carried out the statistical analyses and drafted the manuscript. FAT and ER contributed to the design and revision of the manuscript. KT contributed by designing the questionnaires, acquiring data, designing the study, methodological supervision and revising and drafting the manuscript. All authors read and approved the final manuscript.

## Pre-publication history

The pre-publication history for this paper can be accessed here:

http://www.biomedcentral.com/1471-2458/13/319/prepub

## References

[B1] Homish GG Leonard KE Kearns-Bodkin JN Alcohol use, alcohol problems and depressive symptomatology among newly married couples Drug Alcohol Depend 2006 83 185 192 10.1016/j.drugalcdep.2005.10.017 16337752 PMC1783684

[B2] Hansson H Zetterlind U Åberg-Örbeck K Berglund M Two-year outcome of coping skills training, group support and information for spouses of alcoholics Alcohol Alcohol 2004 39 135 140 10.1093/alcalc/agh024 14998831

[B3] Tempier R Boyer R Lambert J Mosier K Duncan CR Psychological distress among female spouses of male at risk drinkers Alcohol 2006 40 41 49 10.1016/j.alcohol.2006.09.032 17157719

[B4] Dawson DA Grant BF Chou SP The impact of partner alcohol problems on women’s physical and mental health J Stud Alcohol Drugs 2007 68 66 75 17149519 10.15288/jsad.2007.68.66

[B5] Schuckit MA Smith TL Eng MY Kunovac J Women who marry men with alcohol-use disorders Alcohol Clin Exp Res 2002 26 1336 1343 10.1111/j.1530-0277.2002.tb02676.x 12351927

[B6] Jacob T Dunn NJ Leonard K Patterns of alcohol abuse and family stability Alcohol Clin Exp Res 1983 7 382 385 10.1111/j.1530-0277.1983.tb05489.x 6362462

[B7] Kahler CW McCrady BS Epstein EE Sources of distress among women in treatment with their alcoholic partner J Subst Abuse Treat 2003 24 257 265 10.1016/S0740-5472(03)00033-3 12810147

[B8] Cornelius JR Kirisci L Reynolds M Homish GG Clark DB Husband’s SUD is associated with higher levels of co-occurring but not non-co-occurring psychiatric disorders among their wives Addict Behav 2008 33 1231 1234 10.1016/j.addbeh.2008.04.013 18544467 PMC2504023

[B9] Homish GG Leonard KE The drinking partnership and marital satisfaction: The longitudinal influence of discrepant drinking J Consult Clin Psychol 2007 5 43 51 17295562 10.1037/0022-006X.75.1.43 PMC2289776

[B10] Mudar P Leonard KE Soltysinski K Discrepant substance use and marital functioning in newlywed couples J Consult Clin Psychol 2001 69 130 134 11302271 10.1037//0022-006x.69.1.130

[B11] Kiecolt-Glaser JK Newton TL Marriage and health: His and hers Psychol Bull 2001 127 472 503 11439708 10.1037/0033-2909.127.4.472

[B12] Maes HHM Neale MC Kendler KS Hewitt JK Silberg JL Foley DL Meyer JM Rutter M Simonoff E Pickles A Eaves LJ Assortative mating for major psychiatric diagnoses in two population-based samples Psychol Med 1998 28 1389 1401 10.1017/S0033291798007326 9854280

[B13] Tambs K Moderate effects of hearing loss on mental health and subjective well-being: Results from the Nord-Trøndelag Hearing Loss Study Psychosom Med 2004 66 776 782 10.1097/01.psy.0000133328.03596.fb 15385706

[B14] Ewing JA Detecting alcoholism: the CAGE questionnaire JAMA 1984 252 1905 1907 10.1001/jama.1984.03350140051025 6471323

[B15] Zigmond AS Snaith RP The hospital anxiety and depression scale Acta psychiatr scan 1983 67 361 370 10.1111/j.1600-0447.1983.tb09716.x 6880820

[B16] Søgaard AJ Bjelland I Tell GS Røysamb E A comparison of the CONOR Mental Health Index to the HSCL-10 and HADS. Measuring mental health status in the Oslo Health Study and the Nord-Trøndelag Health Study Nor epidemiol 2003 13 279 284

[B17] Fergusson DM Boden JM Horwood LJ Tests of causal links between alcohol abuse or dependence and major depression Arch gen psych 2009 66 260 266 10.1001/archgenpsychiatry.2008.543 19255375

[B18] Kawachi I Berkman LF Social ties and mental health J Urban Health 2001 78 458 467 10.1093/jurban/78.3.458 11564849 PMC3455910

[B19] Kringlen E Torgersen S Cramer V A Norwegian psychiatric epidemiological study Am J Psychiatry 2001 158 1091 1098 10.1176/appi.ajp.158.7.1091 11431231

[B20] Horverak O Bye EK Det Norske Drikkemønsteret: En studie basert på intervjudata fra 1973-2004 2007 2007 Retrieved from http://www.sirus.no/filestore/Import_vedlegg/sirusrap.2.07.pdf22664675

[B21] Dohrenwend BP Levav I Shrout PE Schwartz S Naveh G Link BG Skodal A Stueve A Socioeconomic status and psychiatric disorders: The causation selection issue Science 1992 255 946 952 10.1126/science.1546291 1546291

[B22] Torvik FA Rognmo K Tambs K Alcohol use and mental distress as predictors of non-response in a general population health survey: The HUNT study Soc Psychiatry Psychiatr Epidemiol 2011 10.1007/s00127-011-0387-3 PMC3328681 21544604

[B23] Knudsen AK Hotopf M Skogen JC Overland S Mykletun A The health status of nonparticipants in a population-based health study: the Hordaland Health Study Am J Epidemiol 2010 172 1306 1314 10.1093/aje/kwq257 20843863

[B24] Høyer G Nilssen O Brenn T Schirmer H The Svalbard study 1988-89: A unique setting for validation of self-reported alcohol consumption Addict 1995 90 539 544 10.1111/j.1360-0443.1995.tb02188.x 7773116

[B25] Skog AOJ The collectivity of drinking cultures: A theory of the distribution of alcohol conusmption Br J Addict 1985 80 83 99 10.1111/j.1360-0443.1985.tb05294.x 3856453

[B26] Ask H Rognmo K Torvik FA Røysamb E Tambs K Non-random mating and convergence over time for alcohol consumption, smoking and excercise: The Nord-Trøndelag Health Study Behav genetics 2012 42 354 365 10.1007/s10519-011-9509-7 22005768

